# Effects of parenting interventions on child and caregiver cortisol levels: systematic review and meta-analysis

**DOI:** 10.1186/s12888-020-02777-9

**Published:** 2020-07-15

**Authors:** Rafaela Costa Martins, Cauane Blumenberg, Luciana Tovo-Rodrigues, Andrea Gonzalez, Joseph Murray

**Affiliations:** 1 Human Development and Violence Research Centre (DOVE), Federal University of Pelotas, Rua Marechal Deodoro 1160, Pelotas, RS 96020-220 Brazil; 2 Post-graduate Program in Epidemiology, Federal University of Pelotas, Pelotas, Brazil; 3 Department of Psychiatry & Behavioural Neurosciences, McMaster University, Hamilton, Canada

**Keywords:** Cortisol, Early interventions, Caregiver, Children, Adolescent, Meta-analysis, Systematic review

## Abstract

**Background:**

Nurturing care, in which children are raised in engaging and safe environments, may reduce child stress and shape hypothalamic-pituitary-adrenal axis functioning. Hence, parent-training programs may impact child cortisol levels, as well as behavioral, social and health outcomes. We conducted a systematic review of the impact of parent-training interventions on children’s and caregivers’ cortisol levels, and meta-analyzed the results.

**Methods:**

In January 2020, searches in PubMed, LILACS, ERIC, Web of Science, Scielo, Scopus, PsycNET and POPLINE databases were conducted, and two independent researchers screened the results for eligible studies – randomized trials that assessed the impact of parent-training interventions on child or caregiver cortisol levels. Random effects were used to pool the estimates, separately for children and caregivers, and for children’s morning and evening cortisol levels, as well as change across the day.

**Results:**

A total of 27 eligible studies were found. Data from 19 studies were extracted and included in the meta-analyses, with 18 estimates of child cortisol levels and 5 estimates for caregiver cortisol levels. The pooled effect size (standardized mean difference) for the effects of parent training programs on morning child cortisol was 0.01 (95%CI: − 0.14 to 0.16; I^2^: 47.5%), and for caregivers it was 0.04 (95%CI: − 0.22 to 0.30; I^2^: 0.0%). Similar null results were observed for child evening cortisol and for the slope between morning and evening child cortisol. No evidence of publication bias was found.

**Conclusion:**

Existing evidence shows no effect of parent-training interventions on child or caregiver post-intervention cortisol. Researchers are encouraged to adopt standardized protocols to improve evaluation standards, to test for intervention effects on psychosocial outcomes that are theorized to mediate the effects on biomarkers, and to use additional biomarkers for chronic stress.

## Background

Nurturing care in childhood, combining parental warmth, sensitivity, stimulation, and clear limits enforced without violence, is associated with lifelong benefits for mental health, behavior, human capital, and social adjustment [1–4]. Randomized trials have demonstrated that parent-training programs can increase nurturing care, reduce child maltreatment, and improve children’s outcomes through the life-course [5–7]. For example, trials of the Nurse-Family Partnership program [8], developed in the United States, found that children whose parents received home visits from nurses supporting family planning, competent caring, and healthy behaviors from pregnancy to child age 2 years, had better outcomes in terms of educational achievement [9], reduced antisocial behavior [10], risky sexual behavior [11], substance use [11], child abuse [10], and even reduced mortality [12].

The varied lifelong benefits of parent-training programmes may be underpinned by biological as well as psychological and social change. One potential biological mediator of their effects is change in cortisol levels in children or parents [13]. Cortisol is a hormone from the glucocorticoid family, produced in the adrenal glands and secreted by the hypothalamic-pituitary-adrenal (HPA) axis as an end product in humans. Cortisol is critical in children’s biological development and homeostatic maintenance, inducing appropriate responses to stress, including altered heart rate and immune system response [14]. The body’s natural maintenance process, called homeostasis, is often disrupted by stressors, which induce adaptations in the organism to re-establish stability [15]. However, when an individual is exposed to prolonged and frequent adversity, cerebral resources involved in this biological regulation can be depleted, and dysregulation in the HPA axis may result in biological vulnerability and increased risk of disease. Children experiencing recurrent stress can develop dysregulated cortisol levels, as the HPA axis continues to develop throughout childhood. Consequences of this kind of toxic stress are a major topic of investigation [14, 16]. Observational studies have found strong associations between chronic stress and later mental, skin and cardiovascular diseases, as well as obesity and unhealthy behaviors, such as smoking and alcohol abuse [17–20].

Nurturing care, engaging and stable environments may reduce child stress and improve HPA axis functioning [4]. These benefits may arise either directly (by reducing harsh parenting), or indirectly (by providing a protective buffer in contexts of adverse social environments). Hence, parent-training programs have the potential to influence child cortisol levels, as well as behavioral, social and health endpoints. Altering cortisol regulation may represent an important mechanism by which parent-training programs affect long-term change.

There is some evidence suggesting parent-training interventions may alter cortisol levels. For example, one study carried out in the United States with mothers and newborn infants showed that a home-visiting program, training parents how to cope with caregiving challenges, lowered morning cortisol levels in children after 17 home visits [21]. Another trial in the United States tested the impact of providing caregivers with training sessions about coping with personal issues, supporting children’s regulatory capabilities and managing child behavior, for children in foster care. Results showed that this early intervention reduced children’s stress levels – indicated by morning to evening cortisol [22].

Slopen and colleagues systematically reviewed studies published up to 2012 on the impact of any type of psychosocial intervention – not restricted to parent-training – on child cortisol levels [13]. Nineteen quasi-experiments and randomized trials were included, that evaluated either parent-focused or child-focused interventions. Overall, 18 studies reported significant effects on child cortisol levels and reactivity. However, results were mixed (some showing increases, and others decreases in cortisol levels); the results were not pooled in meta-analysis. Hackman and colleagues meta-analyzed 28 observational studies and 10 intervention studies up to 2017, focusing on parental warmth and its effects on child cortisol levels. They found a small, but long-term, effect of affectionate parenting on children’s HPA axis regulation [23]. The aim of the present study is to conduct an updated systematic review of the impact of all parent-training interventions on both child and carer cortisol levels, restricting the synthesis to the best quality studies (randomized controlled trials), and to meta-analyze the results.

## Methods

We conducted a systematic review of studies that evaluated the impact of parent-training interventions on children’s cortisol levels. We searched PubMed, LILACS, ERIC, Web of Science, Scielo, Scopus, PsycNET and POPLINE databases. The literature search was run on January 2, 2020, without restriction by date of publication. The combinations of terms used were: (cortisol OR HPA OR hypothalamic pituitary adrenal axis OR hypothalamic-pituitary-adrenocortical OR glucocorticoid OR hydrocortisone) AND (parent* intervention OR parent* training OR parent* program OR parent* education OR maternal intervention OR maternal training OR maternal education OR maternal program OR family intervention OR family training OR family education OR family program) AND (randomized controlled trial* OR trial OR intervention OR experiment OR random allocation OR controlled clinical trial OR early intervention OR intervention study). No limits were applied for the search.

Studies must have met the following eligibility criteria to be included in the review: (a) human studies including children between 0 and 18 years of age; (b) trials that allocated their participants to intervention or control group status using randomization (RCTs); (c) the intervention was a parent-training program involving either caregivers only, children and caregivers, or the entire family; (d) the impact of the intervention was tested for the child or carer’s diurnal cortisol levels or reactivity; (e) the results were published in articles, monographs, dissertations, conference papers, thesis, books or chapters (or were available directly from the authors).

The first author conducted the search. Two researchers carried out the process of reading the titles, abstracts and full texts independently and, in case of divergence they tried to reach a consensus. If a disagreement persisted, a third researcher was involved to resolve the inconsistency. The reference lists of selected articles, Slopen’s [13] review, and Hackman’s [23] metanalysis were scrutinized to identify any additional study eligible for inclusion in the analysis. If a study result was published in more than one report (e.g., an article, conference paper, book chapter), only the journal article was included. Also, if the same sample and intervention resulted in more than one journal article, one was selected for inclusion (giving preference to the article that included the entire sample or analysis that was more comparable with the majority of the other included studies).

The following information was extracted for each study: authors, year of publication, country in which the study was conducted, sample size, type of population (e.g., foster care, high risk families), age range of the children, type of parental intervention, duration and frequency of the intervention, type of biological sample collected to measure cortisol (e.g., saliva, urine, hair), type of cortisol measure (diurnal or reactivity), number of times that cortisol was collected at each assessment, cortisol inter- and intraassay, and method used for cortisol levels measurement (e.g., ELISA, radioimunoassay). Results were recorded in terms of cortisol levels at post-intervention for control and interventions groups, if that information was available, as well as the effect size, and the standard deviation/standard error/confidence intervals.

To assess the methodological quality of included studies we used the Jadad Scale, a scoring system for clinical trials [24]. The scale has seven items and studies receive an overall score ranging from 0 (bad) to 5 (good), derived as the sum of the first 5 items (scored 0 or + 1) and the last two questions (scored 0 or − 1). The scale considers blinding, dropouts and randomization. As double blinding is not possible for parent-training interventions, we considered only questions regarding dropouts and randomizations. As such, the range of possible scores for included studies was from 0 to 3 points.

This systematic review was registered in PROSPERO (International Prospective Register of Systematic Reviews) [25] with registration number CRD42019120257. The PRISMA (Preferred Reporting Items for Systematic Reviews and Meta-Analysis) Statement [26] was used to model this manuscript.

Three separate meta-analyses were performed: one for the impact of parent-training programs on children’s and caregivers’ morning cortisol levels, one for the impact of parent-training programs on children’s evening cortisol levels, and one for the difference of morning and evening cortisol levels in children (change through the day). One author extracted the data of the selected articles, as means and standard deviations wherever possible, and then calculated standardized mean differences as the effect size, with respective confidence intervals. The standardized mean difference represents the mean value in post-intervention for the intervention group minus the mean for the control group, expressed in standard deviation units. Whenever the necessary data was had not been published, emails were sent to authors to request the appropriate information.

Some studies reported multiple post-intervention measures of cortisol. For those, a single outcome was selected for use in the meta-analysis, as follows: a) where multiple measures were available during the same day, separate analyses were run for each time of day (morning vs. evening); b) where multiple morning or multiple evening cortisol measures were available across different days, an average was calculated; c) where cortisol measures were available from both before and after a research-applied stressor, the pre-stress measure was selected for morning cortisol meta-analysis and the individual results of the reactivity study was included in a different meta-analysis.

Some studies reported cortisol levels in the logarithmic scale but did not specify which transformation was used (log_10_ or natural). For those studies, we assumed the most common transformation was applied (the natural logarithm). Because this could not be confirmed, we performed a sensitivity analysis assuming log_10_ for the same study.

For each meta-analysis, pooled estimates were obtained using a random effects model. Heterogeneity was calculated using the I-squared statistic. We examined funnel plots and conducted the Egger test for studies of child morning cortisol only, given the small number of studies of caregiver morning cortisol levels and for child evening cortisol levels [27]. All of the analyses were conducted using the *metan* and *metareg* commands in STATA 15.1 (StataCorp., College Station, TX, USA).

## Results

**Fig. 1:**
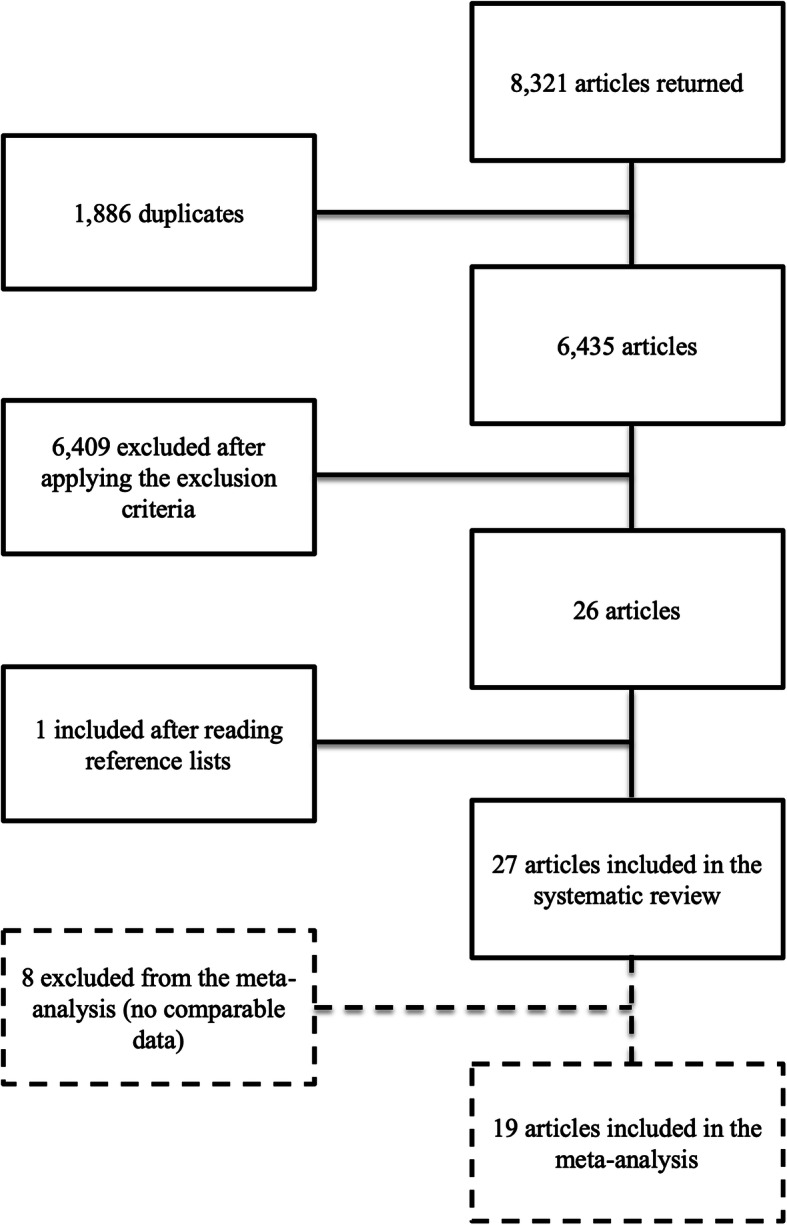
Flowchart showing the selection of articles for the systematic review and the meta-analysis

The search returned 8321 articles, 1886 of which were duplicates (see Fig. 1). After two individuals read the remaining titles and abstracts, 79 articles were selected for reading full texts. Of those, 52 articles were excluded because of the following reasons: 3.9% were review papers; 25.0% were not randomized (or this information was not reported); 7.7% had no intervention; 49.9% were not parent-training interventions; 1.9% did not involve the caregiver in the intervention; 3.9% described a subset of results from an already selected article; 1.9% did not measure cortisol; 5.8% occurred entirely or partially during pregnancy (before children could be involved in the intervention).

A total of 27 studies were identified as eligible for the review. Of these, 19 studies had not been included in the previous review by Slopen et al. [13], and 14 had not been included in the review by Hackman et al. [23]. Of the 27 studies eligible for our review, eight were narratively reviewed but could not be included in a meta-analysis: one because it measured hair cortisol (unlike all others), three because the means or the measures of variance were not available, and four because the study data were not comparable to the others in terms of analyses and types of results.

Of the 19 studies that could be included in the meta-analyses, 14 provided estimates for the impact on cortisol for children only, three provided estimates for both children and caregivers, and one study had results for caregivers only – this last caregiver only study [28] had separate estimates for two different interventions - counting twice in the final number of analyzed studies. Hence, overall, the meta-analyses included 18 estimates for the impact of parent-training interventions on children’s cortisol levels, and 5 estimates for caregivers’ cortisol levels.

Table 1 summarizes the characteristics of the 27 studies included in the review. All but two of the studies were conducted in high-income countries. The sample sizes ranged from 20 to 240 dyads, with few studies (*n* = 4) including over 150 randomized individuals. Most samples of children (*n* = 18) were aged between 1 and 5 years. All of the studies but one collected saliva to measure cortisol. There was little consistency in the time of day that cortisol was measured, and one reactivity study did not report the time or period of the day that cortisol was measured. The length of follow-up after interventions were implemented varied widely too – from weeks to years. Where results from multiple follow-ups were reported in a single study, the results of the first assessment were selected for inclusion in the meta-analysis.

**Table 1:** Summary characteristics of studies included in the systematic review (*n* = 27)

Characteristics	Number of studies (%)
Year of publication	
2000–2009	7 (25.9)
2010–2019	20 (74.1)
Country	
United States	20 (74.1)
Netherlands	3 (11.1)
Argentina	1 (3.7)
Canada	1 (3.7)
Iran	1 (3.7)
Switzerland	1 (3.7)
Sample size	
< 100	14 (51.9)
100 < *n* < 150	9 (33.3)
≥ 150	4 (14.8)
Children’s mean age at baseline	
< 1 year	4 (14.8)
≥ 1 year and < 5 years	18 (66.7)
≥ 5 years	5 (18.5)
Type of population	
High-risk families^a^	6 (22.2)
Foster care/Adopted children	8 (29.7)
Hospitalized-based	3 (11.1)
Maltreated children	3 (11.1)
Caregiver’s death, sick or divorced	4 (14.8)
General population	3 (11.1)
Cortisol outcome	
Saliva (diurnal)	16 (59.3)
Saliva (reactivity)	10 (37.0)
Hair	1 (3.7)
Technique used for biological analysis	
Enzyme immunoassay (EIA)	13 (48.2)
Radioimunoassay	3 (11.1)
Enzyme-linked immunosorbent assay (ELISA)	3 (11.1)
Other techniques	4 (14.8)
Not described	4 (14.8)
Cortisol intraassay < 10%	
Yes	19 (70.4)
No	0 (0.0)
Not described	8 (29.6)
Cortisol interassay < 10%	
Yes	15 (55.6)
No	3 (11.1)
Not described	9 (33.3)

^a^Children from poor families, or with depressed mothers, or mothers that abuse of illegal substances, or in the Child Protective Service records, or sibling of an adjudicated youth

Table 2 describes, for each of the 27 individual studies included in the review, their sample characteristics, details of the intervention, and methodological quality. In terms of the stated goals of the interventions, the great majority of studies evaluated programs aiming to improve affectionate or stimulating parenting, or other positive interaction with children; while others had the stated goal of improving overall child development, socio-emotional adjustment, or reducing externalizing behaviors.

**Table 2:** Characteristics of individual studies included in the systematic review

Authors	Country	Sample size (I:C)	Frequency and duration of intervention	Aim of parental intervention	Cortisol measure	Time of measurements	Number of measurements	Jadad Score
Bakermans-Kranenburg MJ et al. (2008) [29] ^b^	Netherlands	130 (66:64)	6 monthly sessions (1 h30 each)	To stimulate parents’ sensitive interactive skills (focusing on sensitive discipline) to prevent further increase of child externalizing problems	Diurnal	Wake-up, before lunch and bedtime	3	2
Berlin LJ et al. (2019) [30]	United States	153 (76:77)	10 weekly sessions	To provide nurturance, to follow the child’s lead with delight and to avoid intrusive and frightening behaviors	Reactivity	24% before 10 am, 40% 10 am-1 pm, 36% 1 pm or later)	4 (pre-task, 5 min post-task, 20 min post-task, 40 min post-task)	2
Bernard K et al. (2015) [31]^c^	United States	212 (100:112)	10 weekly sessions (1 h each)	To help parents become more synchronous and nurturing, and less frightening, in their interactions with their children	Diurnal	Wake-up and bedtime	2	2
Bernard K et al. (2015) [32] ^c^	United States	101 (56:45)	10 weekly sessions (1 h each)	To help parents become more synchronous and nurturing, and less frightening, in their interactions with their children	Diurnal	Wake-up and bedtime	2 (over 3 days)	2
Bernard K et al. (2015) [33] ^c^	United States	115 (54:61)	10 weekly sessions	To increase resilience to distress, increase synchronous interactions, and decrease frightening parental behavior	Diurnal	30 min after wake-up and bedtime	2 (over 3 days)	3
Borghini A et al. (2009) [34]	Switzerland	80 (40:40)	4 to 5 sessions in a 4-month period (1 h30 each)	To improve the quality of the parent-baby relationships by helping parents to better understand children and support development	Reactivity and diurnal	8 h, 12 h, 14 h, 14 h20, 14 h40, 16 h, 20 h	7	1
Brotman LM et al. (2007) [35]	United States	92 (47:45)	22 weekly sessions (90 min individually + 30 min parent-child each) + 10 biweekly sessions (90 min each) + 6 sessions in a 6 to 8-month period	To improve parenting practices and preschoolers’ social competence with the goal of preventing later conduct problems	Reactivity and diurnal	Morning (40%) and afternoon (60%)	2 (pre-task and post-task) + 4 times a week after (7 am, 12 am, 4 pm, 8 pm)	1
Bugental DB et al. (2010) [21]^d^	United States	147 (69:78)	17 sessions in a 1-year period + possible visits in a 3-year period	To assist parents to acquire cognitive resources (skills in obtaining information relevant to child development, knowledge about effective ways to manage caregiving challenges and ways to obtain information and make contact with community agencies)	Diurnal	Mid-morning (10 am)	1	1
Cicchetti D et al. (2011) [36] ^b^	United States	91 (56:35)	46 weekly sessions	To encourage sensitive interactions by helping parents form positive representations of themselves and the caregiver-child relationship, and to teach parenting skills, relaxation techniques, and behaviors that promote social support	Diurnal	Mid-morning (10 am)	1	1
DePasquale CE et al. (2018) [37]	United States	66 (34:32)	10 weekly sessions	To enhance nurturance and synchrony while reducing frightening behavior in at-risk families	Reactivity	Mid-morning (10 am)	3 (pre-task, 15 min post-test, 30 min post-task)	2
Dozier M et al. (2006) [22]	United States	60 (30:30)	10 weekly sessions (1 h each)	To help caregivers override their own issues that interfere with providing nurturing care, and provide an environment that helps children develop regulatory capabilities	Diurnal	Wake-up and bedtime	2 (over 2 days)	1
Dozier M et al. (2008) [38]	United States	93 (46:47)	10 weekly sessions (1 h each)	To help parents become more synchronous and nurturing, and less frightening, in their interactions with their children	Reactivity	–	3 (pre-task, 15 min post-test, 30 min post-task)	1
Fisher PA et al. (2007) [39]	United States	117 (57:60)	6 to 9 months of intensive training (12 h) + daily calls + weekly sessions for children and parents individually	To address the developmental and social-emotional needs of foster preschoolers	Diurnal	30 min after wake-up and 30 min before bedtime	2 (day 1) + 2 (day 2) for 12 months	1
Fisher PA et al. (2008) [40] ^b^	United States	117 (57:60)	6 to 9 months of intensive training (12 h) + Daily calls + weekly sessions for children and parents individually	To address the developmental and social-emotional needs of foster preschoolers	Diurnal	30 min after wake-up and 30 min before bedtime	2	1
Habersaat S et al. (2014) [41] ^b^	Netherlands	60 (30:30)	1 session (60–80 min) + 3 sessions (10 min)	To enhance parent’s observation and understanding of the specific competencies of their preterm infant and promoting parents’ sensitivity and responsiveness toward the infant’s behavioral characteristics	Diurnal	Wake-up (8 h), before meal at noon, afternoon (17 h), before bedtime (20 h)	4 (over 2 days)	2
Letourneau N et al. (2011) [42]	Canada	60 (27:33)	12 weekly sessions	To teach new mothers about maternal–infant interactions, contingent responsiveness, and to provide support for postpartum depressed mothers	Diurnal	Wake-up, noon, mid-afternoon, bedtime	4	2
Luecken LJ et al. (2010) [43]	United States	139 (78:61)	12 weekly sessions (2 h of interaction + 12 min of discussion)	To increase positive caregiver-child relationships, effective discipline, and to decrease children’s exposure to stressful events	Reactivity	Afternoon/evening (between 3 and 9 pm)	4 (pre-task, post-task, 15 min post-task, 30 min post-task)	1
Luecken LJ et al. (2014) [44] ^b^	United States	139 (78:61)	12 weekly sessions (2 h)	To increase the positive quality of the caregiver-child relationship, enhance caregivers’ use of effective discipline, decrease caregiver mental health problems, decrease children’s exposure to stressful events, improve youth coping skills, and to promote adaptive beliefs about why negative events occur	Reactivity	Pre-task, post-task, 15 min post-task, 30 min post-task	4	2
Luecken LJ et al. (2015) [45]	United States	240 (164:76)	11 group sessions and 2 individual sessions in a 15-year period (1 h45)	To improve mother–child relationship quality and effective discipline, to decrease barriers between the mother and child, and to decrease interparental conflict	Reactivity	Morning and evening	4 (pre-task, post-task, 20 min post-task, 40 min post-task)	2
Nelson EM et al. (2013) [46]	United States	54 (not described)	10 weekly sessions (1.5 h)	To build caregiver’s confidence and competence in sensitivity, to have developmentally-appropriate expectations, and reframe caregiver’s understanding and responses to children’s ambiguous cues and difficult behavior	Reactivity	On arrival, before task, 30 min post-task, 45 min post-task, next morning 30 min after wake-up	5	2
O’Neal CR et al. (2010) [47]	United States	92 (47:45)	22 sessions for parents + 22 sessions for preschoolers + 22 parent-child interactions + 10 home visits	To encourage parents to use nonharsh, consistent, and appropriate disciplinary strategies, be less critical, use positive reinforcement and promote children’s social competence	Diurnal	Majority in the afternoon, but some mid-morning	1	1
Pirnia B et al. (2019) [48] ^b^	Iran	50 (22:28)	12 weekly sessions	To improve communication in children and to practice interactive discipline in parents	Diurnal	Not described	3	2
Poehlmann-Tynan J et al. (2019) [49] ^b^	United States	39 (25:14)	8 weekly sessions (2 h)	To cultivate mindfulness, self-compassion, equanimity and compassion to others, and its applications to parenting	3 cm of hair	NA	NA	2
Prats LM et al. (2018) [50]	Argentina	46 (23:23)	13 weekly sessions (50 min)	To promote cognitive development of children through the promotion of parenting practices	Diurnal	Morning (8 h30 to 9 h) and night	2	2
Toth SL et al. (2015) [28] ^a^	United States	157 (44/34:27)	48 weekly sessions	Intervention I: to improve the mother–child relationship, through the provision of developmental guidance based on maternal concerns.Intervention II: to improve current concerns about parental education, maternal stress, and social support encouraging mothers to seek further education and employment and enhanced informal social support.	Diurnal	Mid-morning (as close as 10 am)	1	1
Turpyn CC et al. (2019) [51] ^b^	United States	20 (10:10)	8 weekly sessions (2 h)	To promote mindfulness intervention, focusing on parenting interactions	Reactivity	Pre-task, post-task, 15 min post-task, 30 min post-task	4	3
Van Andel H et al. (2016) [52]	Netherlands	123 (65:58)	Every 2 weeks in a 3-month period (90 min)	To help foster caregivers interpret the interaction with their child	Diurnal	Wake-up and bedtime	2	2

^a^The study has two different interventions and a control group. ^b^Studies that were included in the systematic review only. ^c^Those are not estimates from the same study, they are different papers published in the same year. ^d^The sample size of the intervention and control groups could not be determined because only the total sample size was reported – based on the total sample size, we estimated the number of individuals in each group using the values of another paper from the same project [53]

All 27 described the intervention as randomized (100.0%), which was required for inclusion in this review, though only two (7.4%) described the randomization method (both studies used an appropriate method). These same two articles reported the randomization method, scoring 3 on the Jadad quality scale (the maximum score possible), but the majority of the articles scored only 2 points (51.9%). Only twelve studies (44.4%) reported on withdrawals and dropouts. The mean number of saliva samples collected was two for diurnal and four for reactivity studies. Regarding the 10 reactivity studies, several stressors were used before the cortisol measurement: a) Strange Situation (*n* = 5); b) Discussion task (*n* = 3); c) Trier Social Stress Task (*n* = 1); d) Social Challenge (*n* = 1).

**Fig. 2:**
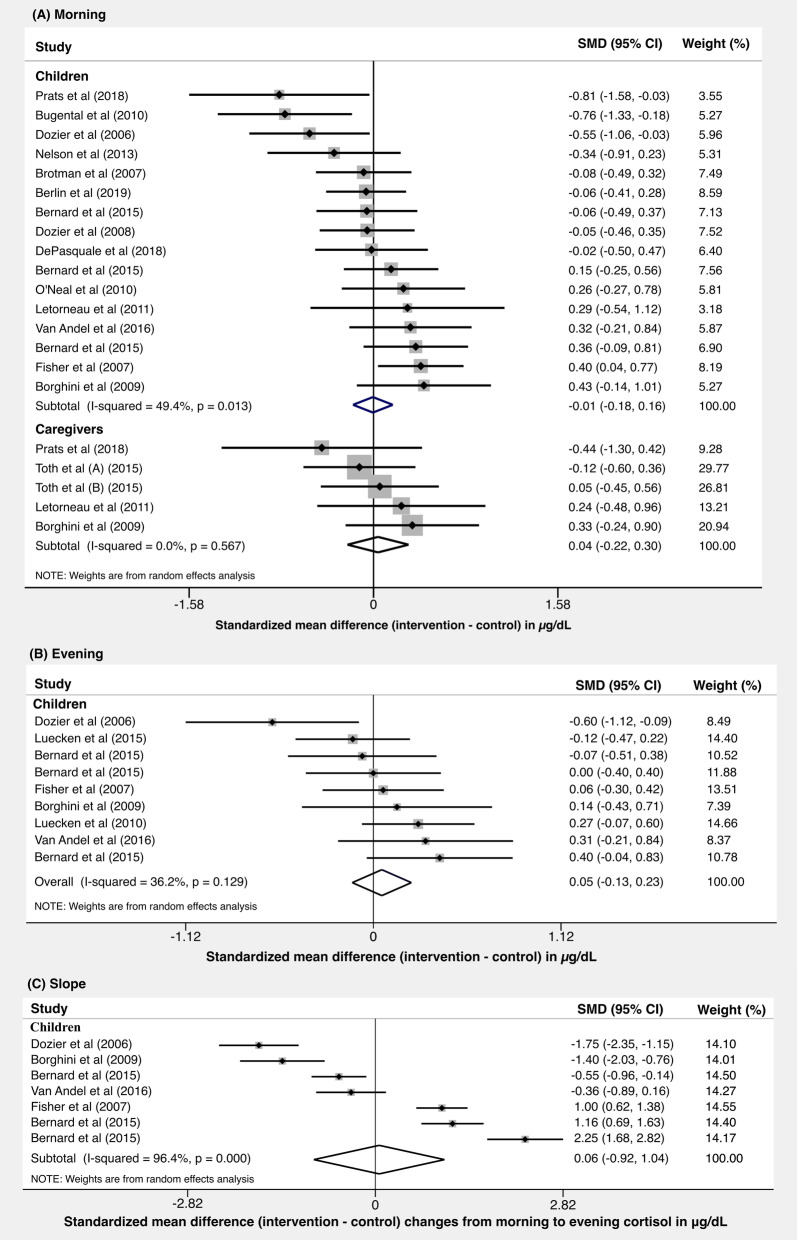
Meta-analysis of the impact of parent-training interventions on cortisol levels in children-caregivers’ dyads in the (**a**) morning, (**b**) evening, and (**c**) changes from morning to evening

Figure 2 presents the meta-analytic results for studies of morning cortisol levels separately for children and caregivers (A), and evening cortisol levels for child cortisol levels (B). Effect sizes represents the standardized mean difference between the values of cortisol in the intervention group and the control group at post-intervention assessment. The pooled effect of morning cortisol levels (Fig. 2a) was nearly zero for both children (0.01; 95%CI: − 0.14 to 0.16; I^2^: 47.5%) and caregivers (0.04; 95%CI: − 0.22 to 0.30; I^2^: 0.0%). The pooled effect of evening cortisol levels for children (Fig. 2b) was also nearly zero (d = 0.04; 95%CI: − 0.18 to 0.26; I^2^: 39.7%). No meta-analysis of evening levels for caregivers was possible, given only one study reported results from this outcome. Figure 2c shows results for the effect of the parental interventions on changes in child cortisol levels across the day (morning minus evening values). The pooled effect for this change across the day was 0.06 (95%CI, − 0.92 to 1.04; I^2^: 96.4%). The individual means and standard deviations used to calculate effect sizes for the meta-analyses can be found in supplementary Tables 1 and 2.

**Fig. 3:**
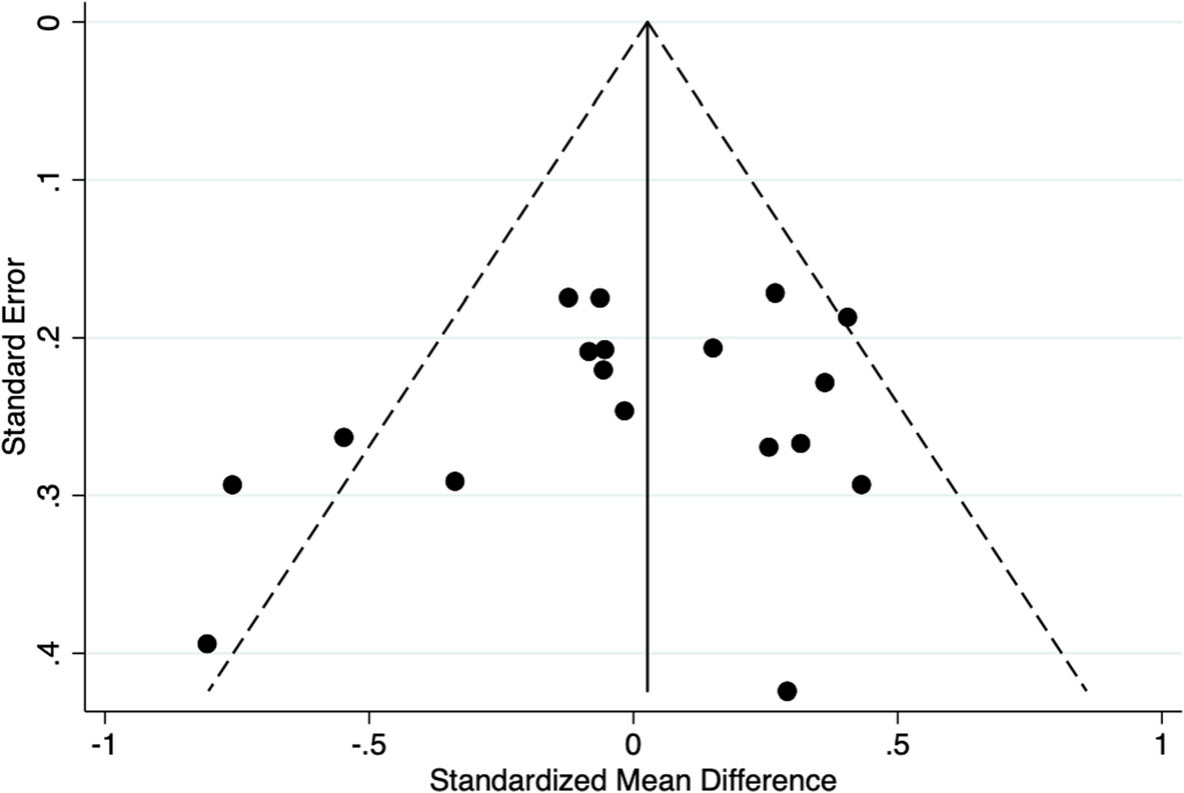
Funnel plot: standardized mean differences between intervention and control group in post-intervention child morning cortisol levels

We conducted sensitivity analysis for results on children’s morning cortisol levels (for which the most studies were available). Re-running these analyses, instead of assuming that studies used a natural log for cortisol measures, where the scale was not actually reported, we used instead a log_10_ scale to calculate the effect sizes, but this showed no difference in the results. As shown in Fig. 3 we found no evidence of publication bias, analysing children’s morning cortisol results (Egger test *p*-value > 0.05).

We used meta-regression to test if the age of the children at baseline (< 1 year, ≥ 1 year and < 5 years, ≥ 5 years) would modify the results. No difference was found (morning *p* = 0.15, evening: *p* = 0.95; across the day: *p* = 0.33). The specific time of data collection, both for morning and evening cortisol data collection, differed across the studies. As such, we used meta-regression to test if the time at which cortisol was collected in the morning (immediately after wake-up, 30 min after wake-up, mid-morning, or morning and afternoon in the same study) or in the evening (afternoon and evening in the same study, 8 pm, 30 min before bedtime, or at bedtime) influenced the estimate of the impact of parenting interventions on child cortisol level. No difference was found across these time categories (morning *p* = 0.59; evening *p* = 0.89).

**Fig. 4:**
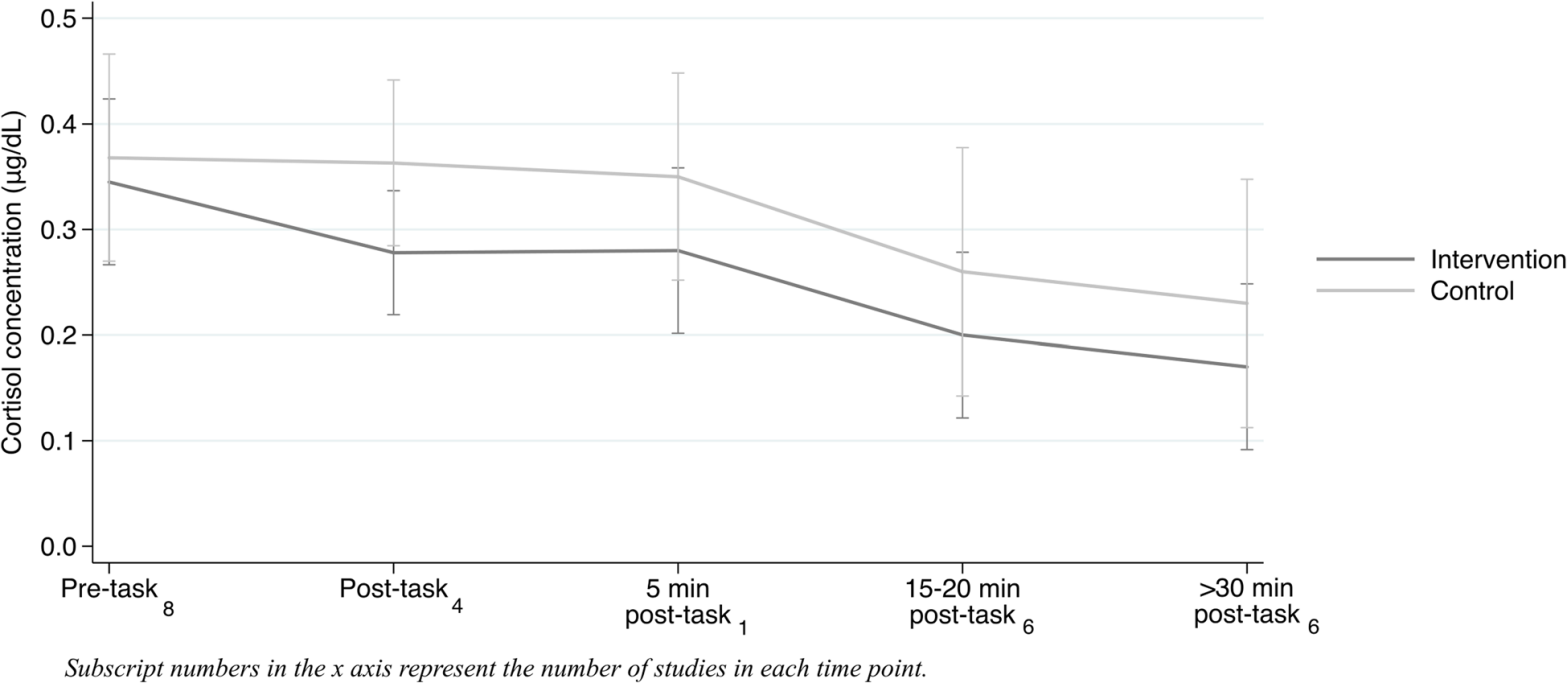
Intervention and control group levels of cortisol concentration before and after stress tasks in reactivity studies

Figure 4 shows the results for the eight studies that examined effects of parent-training on child cortisol reactivity. The graph shows the average level of cortisol, separately for intervention and control groups, prior to children being exposed to a stressor task, immediately post-task, and 5, 15–20, and > 30 min later. As can be seen, from relatively similar pre-intervention levels, there is some divergence in post-intervention measures – with the intervention group showing slightly lower values compared to the control group, but all differences were not significant (*p* > 0.22 in all periods of saliva collection).

Considering the eight studies that were eligible for the current review, but could not be included the meta-analyses, findings were also mixed. In one study, cortisol levels were reduced, but only for children with the *DRD4* 7-repeat allele, a gene that moderated the effects of parenting-training on cortisol [29]. Three studies found no differences [36, 44, 51]. Habersaat found that the control group had lower cortisol levels than the intervention group in two post-intervention assessments [41]. In the study by Fisher and colleagues, there was a decrease in the mean cortisol level of the caregivers who participated in the intervention [40]. One other study measured hair cortisol in children and the results showed that the cortisol concentration was lower in the intervention group compared with the controls [49]. Because hair cortisol measures evaluate the average level of cortisol over several months prior to the sample being taken, these results were not comparable to the other studies included in the review (based on saliva and urine samples), and so could not be included in the meta-analysis [48].

## Discussion

We meta-analyzed the impact of parent-training interventions on child and caregiver cortisol levels. The results showed no discernible effect of parenting interventions on either outcome. This is an interesting null finding considering the extent of evidence for social and health benefits achieved by numerous parent-training programs [5, 12, 54–56]. If parenting interventions affect other outcomes through the life-course, but not cortisol, then other social, psychological or biological mechanisms must explain those changes. For example, other mediators of the effects of parent-training programs on child antisocial behavior might be discipline strategies and child learning processes, or other parenting skills that do not necessarily affect cortisol levels [57, 58].

Slopen and colleagues [13] previously reviewed studies published on the impact of psychosocial interventions on child cortisol regulation up to 2012, with little consistency in the overall results. In a meta-analysis on parental warmth an cortisol, Hackman [23] also found inconsistent and overall null results among 28 prospective and 10 intervention studies up to 2017. The current review provides an updated meta-analysis focusing on parent-training programs, quantitatively pooling estimates across 18 intervention studies of child cortisol, and 5 studies of caregiver cortisol. The lack of average effects for children or their caregivers in the current review, and high variability in the results, are thus consistent with findings by Slopen et al. [13] and Hackman et al. [23].

One possible reason for why no effect on cortisol was observed in the current review would be if the interventions had not achieved more proximal changes in parent-child relationships or caregiving environments, that are required to subsequently change cortisol levels. Among the 27 studies reviewed here, only three [21, 30, 44] reported tests of the intervention on parenting practices, and all reported positive results. Two studies found that parenting significantly mediated intervention effects on child cortisol, and one found a significant effect of the intervention on parenting practices, although no association was found between parenting and cortisol in that study. However, for all the other studies reviewed here, it is not clear if the interventions influenced caregivers and their parenting practices in a way that could subsequently have affected child cortisol. As such, it remains possible that no overall effects on cortisol were found in this meta-analysis because the interventions evaluated here were generally ineffective regarding more proximal parenting processes. Note that if this were the case, possibly other interventions, known to be more effective at changing parenting practices, would show cortisol effects.

Another possible explanation for the null results found in this meta-analysis is the low quality of many of the included studies. Generally, most had small samples and reporting on a number of methodological procedures was often poor, such that it is not clear, for example, whether adequate randomization methods were used; reporting on a number of methodological procedures was also often poor. In some studies it was clear that randomization had not succeeded in balancing intervention and control groups on baseline cortisol levels, and some studies did not report baseline values, which could have biased the results. Additionally, few studies reported that the analyses were conducted on an intention to treat basis.

It is also possible that the method by which cortisol was measured explains the varying individual study findings and null pooled effects. All but one study used saliva samples, which provide measures of acute cortisol levels (in terms of minutes), and are influenced by many possible concurrent factors, such as circadian rhythm, time of awakening and sleep patterns (e.g., naps for children), use of medicine, and exercise, as well as possible intervention effects. It is important to highlight that none of the studies evaluated in our review took into account other challenges that might have been present on the day cortisol was measured. Evidence suggests that difficult days are associated with higher levels of cortisol. For example, a study showed that losing an important soccer match had an impact on cortisol, elevating the concentration in saliva [59]. Among children recently starting childcare, higher levels of cortisol have been found than among children spending the day at home [60]. Thus, when saliva or other measures of acute cortisol are used, questionnaires evaluating perceived stress of the day when cortisol is collected is particularly important.

Saliva samples, unless repeated many times over weeks or months, do not measure the type of “toxic stress” that is considered most damaging to development, and which parenting interventions may hope to influence. A new technique to obtain more relevant measures of chronic stress is to collect cortisol from hair samples. That approach, a proxy measure of the HPA axis activity over the previous months, evaluates chronic stress, which is less influenced by immediate variables, and may better capture parenting intervention effects not seen in the studies reviewed here [61, 62]. The single study using this method found that parent-training intervention can reduce chronic cortisol levels [49]. The use of markers like hair samples of cortisol in future studies might better represent the chronic stress that is considered so toxic for human functioning and development.

Even though the results of the current meta-analyses were null, the effectiveness of parent-training interventions on other important parent and child outcomes is well established. For example, parenting programs have been shown to reduce parental self-reported stress [63]; lower child internalizing [64] and externalizing [65] behaviors; reduce sleep problems [66] and excessive crying [67]. As such, even if parent-training programs do not affect acute cortisol levels, they are still important for improving other health and social outcomes [67, 68].

Other biological markers of intervention effects, including more studies on chronic levels of cortisol, should be investigated in future studies. In this review we focus on HPA axis function because it is the most widely studied mechanism explaining associations between early adversity and child outcome; however other viable candidates remain largely unexplored, specifically, inflammatory markers, neural structure and function and epigenetic modifications. These four factors have been identified as likely mediators of the biological embedding of early experiences and should be studied in future as modifiable factors within intervention programs [69].

The following limitations should be considered in relation to this review. Although the literature on cortisol outcomes following parent-training program is growing, we were still limited to meta-analyzing a relatively small number of primary studies. Although we tried to increase homogeneity across studies by selecting more comparable measures, cortisol is a hormone with varying patterns through early morning, mid-morning, late morning, afternoon and evening, and as the exact timing of the biological sample collection was different between studies, this could affect the results. There was also considerable variability between studies in the type of population that was sampled, the type, intensity and duration of the parent-training intervention, and other methodological characteristics (children’s age, length of follow-up, type of cortisol outcome) that could have affected the results. Although ecological validity achieved by testing across multiple days and using a mix of weekdays and weekends is important, studies typically do not do that because of the burden to participants and expense of processing saliva samples – most studies in this review only evaluated children on 1 or 2 days.

## Conclusion

This meta-analysis found no effect of parent-training interventions on child or caregivers cortisol levels. There was no evidence of publication bias. However, researchers are encouraged to adopt standardized protocols to evaluate the effects of parent-training programs on cortisol mediated by parental practices, and also to use additional biomarkers for chronic stress that are less influenced by other variables.

## Supplementary information

**Additional file 1: Table S1.** Descriptive values of the first sample of child cortisol post-intervention – included in the metanalysis. **Table S2.** Descriptive values of the first sample of caregiver cortisol post-intervention – included in the metanalysis.

## Data Availability

Not applicable.

## Abbreviation

HPA: Hypothalamic-pituitary-adrenal
