# Supplementary material for: Effects of parenting interventions on child and caregiver cortisol levels: systematic review and meta-analysis
Source: BMC Psychiatry. 2020 Jul 15;20:370. doi: 10.1186/s12888-020-02777-9 (PMC7362449; doi:10.1186/s12888-020-02777-9)
Supplement: Supplementary file 1 — Additional file 1: Table S1. Descriptive values of the first sample of child cortisol post-intervention – included in the metanalysis. Table S2. Descriptive values of the first sample of caregiver cortisol post-intervention – included in the metanalysis. [file 12888_2020_2777_MOESM1_ESM.docx]

Supplementary table 1. Descriptive values of first sample of cortisol from the articles measuring children post-intervention cortisol included in the metanalysis

| **Author (Year)** | **Intervention** | | | | | **Control** | | | | |
| --- | --- | --- | --- | --- | --- | --- | --- | --- | --- | --- |
|  | **N** | **Mean^#^** | **SD^#^** | **Log-transformed Mean^#^** | **Log-transformed SD^#^** | **N** | **Mean^#^** | **SD^#^** | **Log-transformed Mean^#^** | **Log-transformed SD^#^** |
| Morning |  |  |  |  |  |  |  |  |  |  |
| Berlin et al (2019) | 64 | 0.46 | 0.78 | -0.78 | -0.25 | 67 | 0.51 | 0.8 | -0.67 | -0.22 |
| Bernard et al (2015)^&^ | 39 | 0.24 | 0.18 | -1.43 | -1.71 | 39 | 0.18 | 0.15 | -1.71 | -1.90 |
| Bernard et al (2015)^&^ | 45 | 0.22 | 0.18 | -1.51 | -1.71 | 38 | 0.23 | 0.17 | -1.47 | -1.77 |
| Bernard et al (2015)^&^ | 43 | 0.20 | 0.17 | -1.63 | -1.75 | 52 | 0.17 | 0.19 | -1.77 | -1.69 |
| Borghini et al (2009) | 22 | 0.62 | 0.80 | -0.47 | -0.22 | 26 | 0.36 | 0.35 | -1.01 | -1.05 |
| Brotman et al (2007) | 47 | 1.27 | 2.44 | 0.24 | 0.89 | 45 | 1.57 | 4.38 | 0.45 | 1.48 |
| Bugental et al (2010) | 25 | 0.11 | 0.05 | -2.21 | -3.00 | 25 | 0.17 | 0.1 | -1.77 | -2.30 |
| DePasquale et al (2018) | 34 | 0.08 | 1.86 | -2.53 | 0.62 | 32 | 0.12 | 2.29 | -2.16 | 0.83 |
| Dozier et al (2008) | 46 | 0.42 | 1.61 | -0.87 | 0.47 | 47 | 0.51 | 1.62 | -0.68 | 0.48 |
| Dozier et al (2006) | 30 | 0.41 | 0.43 | -0.89 | -0.84 | 30 | 0.80 | 0.91 | -0.22 | -0.09 |
| Fisher et al (2007) | 57 | 0.49 | 0.22 | -0.71 | -1.51 | 60 | 0.39 | 0.27 | -0.94 | -1.31 |
| Letorneau et al (2011) | 9 | 2.72 | 1.68 | 1.00 | 0.52 | 15 | 2.33 | 1.1 | 0.85 | 0.10 |
| Luecken et al (2015) | 115 | 0.09 | 0.05 | -2.45 | -3.06 | 46 | 0.09 | 0.05 | -2.39 | -3.06 |
| Luecken et al (2010) | 78 | 0.12 | 0.09 | -2.16 | -2.41 | 61 | 0.09 | 0.06 | -2.36 | -2.81 |
| Nelson et al (2013) | 23 | 0.07 | 0.06 | -2.70 | -2.81 | 25 | 0.12 | 0.19 | -2.15 | -1.64 |
| O'Neal et al (2010) | 33 | 1.31 | 1.65 | 0.27 | 0.50 | 24 | 0.91 | 1.39 | -0.09 | 0.33 |
| Prats et al (2018) | 19 | 0.24 | 0.07 | -1.42 | -2.74 | 11 | 0.30 | 0.08 | -1.21 | -2.48 |
| Van Andel et al (2016) | 30 | 0.31 | 0.23 | -1.17 | -1.47 | 27 | 0.25 | 0.15 | -1.39 | -1.88 |
| Evening |  |  |  |  |  |  |  |  |  |  |
| Bernard et al (2015) | 39 | 0.15 | 0.14 | -1.90 | -1.97 | 39 | 0.16 | 0.16 | -1.83 | -1.83 |
| Bernard et al (2015) | 45 | 0.15 | 0.16 | -1.90 | -1.83 | 38 | 0.09 | 0.14 | -2.41 | -1.97 |
| Bernard et al (2015) | 43 | 0.12 | 0.15 | -2.12 | -1.90 | 52 | 0.12 | 0.11 | -2.12 | -2.21 |
| Borghini et al (2009) | 22 | 0.25 | 0.56 | -1.39 | -0.58 | 26 | 0.19 | 0.26 | -1.66 | -1.35 |
| Dozier et al (2006) | 30 | 0.12 | 0.13 | -2.12 | -2.04 | 30 | 0.42 | 0.69 | -0.87 | -0.37 |
| Fisher et al (2007) | 57 | 0.11 | 0.19 | -2.21 | -1.66 | 60 | 0.10 | 0.15 | -2.30 | -1.90 |
| Van Andel et al (2016) | 30 | 0.15 | 0.34 | -1.90 | -1.08 | 27 | 0.07 | 0.10 | -2.66 | -2.30 |
| Slope |  |  |  |  |  |  |  |  |  |  |
| Bernard et al (2015) | 39 | 0.29 | 0.08 | -1.24 | -2.53 | 39 | 0.29 | 0.04 | -1.24 | -3.22 |
| Bernard et al (2015) | 45 | 0.38 | 0.04 | -0.97 | -3.22 | 38 | 0.18 | 0.08 | -1.72 | -2.53 |
| Bernard et al (2015) | 43 | 0.37 | 0.21 | -0.99 | -1.56 | 52 | 0.38 | 0.21 | -0.97 | -1.56 |
| Borghini et al (2009) | 22 | 0.03 | 0.03 | -3.51 | -3.51 | 26 | 0.08 | 0.04 | -2.53 | -3.22 |
| Dozier et al (2006) | 30 | 0.07 | 0.04 | -2.66 | -3.22 | 30 | 0.14 | 0.04 | -1.97 | -3.22 |
| Fisher et al (2007) | 57 | 0.08 | 0.03 | -2.53 | -3.51 | 60 | 0.05 | 0.03 | -3.00 | -3.51 |
| Van Andel et al (2016) | 30 | 0.16 | 0.07 | -1.83 | -2.66 | 27 | 0.18 | 0.03 | -1.72 | -3.51 |

N: sample size; SD: standard deviation; ^&^Those are not estimates from the same study, they are different papers published in the same year; **^#^**μg/dL

Supplementary table 2. Descriptive values of first sample of cortisol from the articles measuring caregiver’s post-intervention cortisol included in the metanalysis

| **Author (Year)** | **Intervention** | | | | | **Control** | | | | |
| --- | --- | --- | --- | --- | --- | --- | --- | --- | --- | --- |
|  | **N** | **Mean^#^** | **SD** | **Log-transformed Mean** | **Log-transformed SD** | **N** | **Mean^#^** | **SD** | **Log-transformed Mean** | **Log-transformed SD** |
| Morning |  |  |  |  |  |  |  |  |  |  |
| Borghini et al (2009) | 22 | 0.43 | 0.31 | -0.85 | -1.17 | 26 | 0.33 | 0.28 | -1.10 | -1.29 |
| Letourneau et al (2011) | 14 | 2.73 | 1.97 | 1.00 | 0.68 | 16 | 2.31 | 1.56 | 0.84 | 0.44 |
| Prats et al (2018) | 16 | 0.39 | 0.14 | -0.95 | -1.98 | 8 | 0.47 | 0.25 | -0.76 | -1.37 |
| Toth et al (2015) | 44 | 0.30 | 0.14 | -1.20 | -1.96 | 27 | 0.32 | 0.20 | -1.14 | -1.61 |
| Toth et al (2015) | 34 | 0.33 | 0.17 | -1.11 | -1.75 | 27 | 0.32 | 0.20 | -1.14 | -1.61 |
| Evening |  |  |  |  |  |  |  |  |  |  |
| Borghini et al (2009) | 22 | 0.08 | 0.08 | -2.53 | -2.53 | 26 | 0.06 | 0.06 | -2.81 | -2.81 |
| Slope |  |  |  |  |  |  |  |  |  |  |
| Borghini et al (2009) | 22 | 0.35 | 0.09 | -1.05 | -2.41 | 26 | 0.27 | 0.07 | -1.31 | -2.66 |

N: sample size; SD: standard deviation; **^#^**μg/dL
